# Supplementary material for: The role of the angular gyrus in semantic cognition: a synthesis of five functional neuroimaging studies
Source: Brain Struct Funct. 2022 Apr 27;228(1):273–91. doi: 10.1007/s00429-022-02493-y (PMC9813249; doi:10.1007/s00429-022-02493-y)
Supplement: Supplementary file 1 — (PDF 433 kb) [file 429_2022_2493_MOESM1_ESM.pdf]

Supplementary Material

"The role of the angular gyrus in semantic cognition – A synthesis of five functional neuroimaging studies"

(Kuhnke, Chapman et al. 2022 BSF)

Supplementary Results

Behavioral analyses

The following tables report the results of statistical analyses on the behavioral measures, i.e. error rates and mean response times for correct trials. For statistical inference, we first conducted repeated-measures ANOVAs. Significant interactions were resolved using post-hoc paired t-tests. Bold font highlights significant effects ($p < 0.05$ Bonferroni-Holm corrected for multiple comparisons); italic fonts highlights trends ($p < 0.05$ uncorrected).

Table S1. Study A (Chapman and Hartwigsen 2021): Repeated-measures ANOVA.

Effect	df	F	p	partial η^2
**Error Rates**				
TASK	1,39	8.595	**0.006**	0.181
CONDITION	2,78	13.935	**<0.001**	0.263
TASK x CONDITION	2,78	8.881	**<0.001**	0.185
**Mean RTs**				
TASK	1,39	11.652	**0.002**	0.230
CONDITION	2,78	9.853	**<0.001**	0.202
TASK x CONDITION	2,78	10.293	**<0.001**	0.209

Table S2. Study A (Chapman and Hartwigsen 2021): Post-hoc paired t-tests.

Comparison	Mean	SE	T	p (unc.)	p (corr.)
**Error Rates**					
Taxonomic vs. thematic task	-2.219	0.757	-2.932	**0.006**	**0.042**
Taxonomic vs. scrambled task	-0.722	0.653	-1.106	0.276	0.729
Thematic vs. scrambled task	1.496	0.772	1.938	0.060	0.240
Taxonomic task: Taxonomic vs. thematic	3.578	1.150	3.111	**0.003**	**0.024**
Taxonomic task: Taxonomic vs. unrelated	6.995	1.194	5.860	**<0.001**	**<0.001**
Taxonomic task: Thematic vs. unrelated	3.417	0.662	5.165	**<0.001**	**<0.001**
Thematic task: Taxonomic vs. thematic	-3.110	1.275	-2.440	*0.019*	*0.095*
Thematic task: Taxonomic vs. unrelated	0.963	1.349	0.714	0.479	0.552
Thematic task: Thematic vs. unrelated	4.073	1.501	2.713	*0.010*	*0.060*

Scrambled task: Congruent vs. incongruent	0.990	0.834	1.187	0.243	0.729
**Mean RTs**					
Taxonomic vs. thematic task	-82.321	24.117	-3.413	**0.002**	**0.010**
Taxonomic vs. scrambled task	-350.935	39.198	-8.953	**<0.001**	**<0.001**
Thematic vs. scrambled task	-268.613	43.922	-6.116	**<0.001**	**<0.001**
Taxonomic task: Taxonomic vs. thematic	-48.785	25.632	-1.903	0.064	0.256
Taxonomic task: Taxonomic vs. unrelated	101.348	25.950	3.905	**<0.001**	**0.002**
Taxonomic task: Thematic vs. unrelated	150.134	20.793	7.220	**<0.001**	**<0.001**
Thematic task: Taxonomic vs. thematic	0.065	27.350	0.002	0.998	1.000
Thematic task: Taxonomic vs. unrelated	-10.509	24.008	-0.438	0.664	1.000
Thematic task: Thematic vs. unrelated	-10.574	20.461	-0.517	0.608	1.000
Scrambled task: Congruent vs. incongruent	276.798	45.196	6.124	**<0.001**	**<0.001**

Table S3. Study B (Graessner et al. 2021): Repeated-measures ANOVA.

**Effect**	**df**	**F**	**p**	**partial η^2**
**Error Rates**				
TASK	1,32	4.668	**0.038**	0.127
CONDITION	3,96	19.856	**<0.001**	0.383
TASK x CONDITION	3,96	24.287	**<0.001**	0.431
**Mean RTs**				
TASK	1,32	10.462	**0.003**	0.246
CONDITION	3,96	46.066	**<0.001**	0.590
TASK x CONDITION	3,96	61.417	**<0.001**	0.657

Table S4. Study B (Graessner et al. 2021): Post-hoc paired t-tests.

**Comparison**	**Mean**	**SE**	**T**	**p (unc.)**	**p (corr.)**
**Error Rates**					
Implicit task: Meaningful vs. anomalous	-5.195	0.940	-5.525	**<0.001**	**<0.001**
Implicit task: Meaningful vs. pseudoword	-6.223	0.808	-7.701	**<0.001**	**<0.001**
Implicit task: Meaningful vs. single words	1.677	0.527	3.185	**0.003**	**0.021**
Implicit task: Anomalous vs. pseudoword	-1.028	1.096	-0.938	0.355	1.000
Implicit task: Anomalous vs. single words	6.872	1.008	6.820	**<0.001**	**<0.001**
Implicit task: Pseudoword vs. single words	7.900	0.920	8.591	**<0.001**	**<0.001**
Explicit task: Meaningful vs. anomalous	0.487	1.097	0.444	0.660	1.000
Explicit task: Meaningful vs. pseudoword	2.435	0.840	2.899	**0.007**	**0.035**
Explicit task: Meaningful vs. single words	2.922	0.899	3.250	**0.003**	**0.021**
Explicit task: Anomalous vs. pseudoword	1.948	0.908	2.147	*0.040*	*0.160*
Explicit task: Anomalous vs. single words	2.435	0.663	3.672	**0.001**	**0.008**
Explicit task: Pseudoword vs. single words	0.487	0.726	0.671	0.507	1.000
**Mean RTs**					
Implicit task: Meaningful vs. anomalous	-73.727	7.755	-9.507	**<0.001**	**<0.001**
Implicit task: Meaningful vs. pseudoword	-208.421	14.163	-14.716	**<0.001**	**<0.001**
Implicit task: Meaningful vs. single words	-186.626	18.860	-9.895	**<0.001**	**<0.001**

Implicit task: Anomalous vs. pseudoword	-134.694	14.411	-9.346	**<0.001**	**<0.001**
Implicit task: Anomalous vs. single words	-112.899	20.350	-5.548	**<0.001**	**<0.001**
Implicit task: Pseudoword vs. single words	21.795	16.876	1.291	0.206	0.206
Explicit task: Meaningful vs. anomalous	-72.085	14.375	-5.015	**<0.001**	**<0.001**
Explicit task: Meaningful vs. pseudoword	11.511	13.749	0.837	0.409	0.409
Explicit task: Meaningful vs. single words	-64.077	13.898	-4.610	**<0.001**	**<0.001**
Explicit task: Anomalous vs. pseudoword	83.595	12.563	6.654	**<0.001**	**<0.001**
Explicit task: Anomalous vs. single words	8.008	14.153	0.566	0.575	0.575
Explicit task: Pseudoword vs. single words	-75.588	11.968	-6.316	**<0.001**	**<0.001**

Table S5. Study C (Kuhnke et al. 2020): Repeated-measures ANOVA.

**Effect**	**df**	**F**	**p**	**partial η^2**
**Error Rates**				
TASK	2,78	58.110	**<0.001**	**0.598**
SOUND	1,39	10.779	**0.002**	**0.217**
ACTION	1,39	16.109	**<0.001**	**0.292**
TASK x SOUND	2,78	40.548	**<0.001**	**0.510**
TASK x ACTION	2,78	8.432	**0.003**	**0.178**
SOUND x ACTION	1,39	112.272	**<0.001**	**0.742**
TASK x SOUND x ACTION	2,78	35.047	**<0.001**	**0.473**
**Mean RTs**				
TASK	2,78	148.434	**<0.001**	**0.792**
SOUND	1,39	6.550	**0.014**	**0.144**
ACTION	1,39	26.038	**<0.001**	**0.400**
TASK x SOUND	2,78	0.333	0.678	0.008
TASK x ACTION	2,78	7.761	**0.004**	**0.166**
SOUND x ACTION	1,39	169.427	**<0.001**	**0.813**
TASK x SOUND x ACTION	2,78	71.260	**<0.001**	**0.646**

Table S6. Study C (Kuhnke et al. 2020): Post-hoc paired t-tests.

**Comparison**	**Mean**	**SE**	**T**	**p (unc.)**	**p (corr.)**
**Error Rates**					
Lexical decisions: Words vs. Pseudowords	0.625	0.356	1.756	0.087	0.783
Lexical decisions: High sound-high action vs. high sound-low action	0.000	0.684	0.000	1.000	1.000
Lexical decisions: High sound-high action vs. low sound-high action	-0.417	0.512	-0.813	0.421	1.000
Lexical decisions: High sound-high action vs. low sound-low action	-0.729	0.475	-1.535	0.133	1.000
Lexical decisions: High sound-low action vs. low sound-high action	-0.417	0.616	-0.676	0.503	1.000
Lexical decisions: High sound-low action vs. low sound-low action	-0.729	0.729	-1.000	0.323	1.000
Lexical decisions: Low sound-high action vs. low sound-low action	-0.313	0.665	-0.470	0.641	1.000
Sound judgments: High sound-high action vs. high sound-low action	-19.375	2.093	-9.257	**<0.001**	**<0.001**
Sound judgments: High sound-high action vs. low sound-high action	-1.406	1.110	-1.267	0.213	1.000

Sound judgments: High sound-high action vs. low sound-low action	2.292	0.934	2.453	*0.019*	*0.190*
Sound judgments: High sound-low action vs. low sound-high action	17.969	2.688	6.684	**<0.001**	**<0.001**
Sound judgments: High sound-low action vs. low sound-low action	21.667	2.750	7.880	**<0.001**	**<0.001**
Sound judgments: Low sound-high action vs. low sound-low action	3.698	0.788	4.695	**<0.001**	**<0.001**
Action judgments: High sound-high action vs. high sound-low action	-10.104	1.952	-5.177	**<0.001**	**<0.001**
Action judgments: High sound-high action vs. low sound-high action	-12.604	1.663	-7.580	**<0.001**	**<0.001**
Action judgments: High sound-high action vs. low sound-low action	-4.896	1.922	-2.548	*0.015*	*0.165*
Action judgments: High sound-low action vs. low sound-high action	-2.500	2.579	-0.969	0.338	1.000
Action judgments: High sound-low action vs. low sound-low action	5.208	1.303	3.998	**<0.001**	**<0.001**
Action judgments: Low sound-high action vs. low sound-low action	7.708	2.959	2.605	*0.013*	*0.156*
**Mean RTs**					
Lexical decisions: Words vs. Pseudowords	-117.999	11.131	-10.600	**<0.001**	**<0.001**
Lexical decisions: High sound-high action vs. high sound-low action	7.141	4.630	1.542	0.131	0.656
Lexical decisions: High sound-high action vs. low sound-high action	1.265	4.788	0.264	0.793	1.000
Lexical decisions: High sound-high action vs. low sound-low action	-18.302	5.139	-3.562	**0.001**	**0.007**
Lexical decisions: High sound-low action vs. low sound-high action	-5.876	4.564	-1.287	0.206	0.822
Lexical decisions: High sound-low action vs. low sound-low action	-25.443	4.447	-5.721	**<0.001**	**<0.001**
Lexical decisions: Low sound-high action vs. low sound-low action	-19.567	4.762	-4.109	**<0.001**	**0.002**
Sound judgments: High sound-high action vs. high sound-low action	-138.208	12.006	-11.511	**<0.001**	**<0.001**
Sound judgments: High sound-high action vs. low sound-high action	-116.031	18.584	-6.244	**<0.001**	**<0.001**
Sound judgments: High sound-high action vs. low sound-low action	-60.796	16.696	-3.641	**0.001**	**0.006**
Sound judgments: High sound-low action vs. low sound-high action	22.177	19.704	1.126	0.267	0.822
Sound judgments: High sound-low action vs. low sound-low action	77.412	18.638	4.154	**<0.001**	**0.002**
Sound judgments: Low sound-high action vs. low sound-low action	55.235	9.904	5.577	**<0.001**	**<0.001**
Action judgments: High sound-high action vs. high sound-low action	-142.914	22.543	-6.340	**<0.001**	**<0.001**
Action judgments: High sound-high action vs. low sound-high action	-94.800	14.179	-6.686	**<0.001**	**<0.001**
Action judgments: High sound-high action vs. low sound-low action	-97.251	23.141	-4.203	**<0.001**	**0.001**
Action judgments: High sound-low action vs. low sound-high action	48.114	21.937	2.193	*0.034*	0.206

Action judgments: High sound-low action vs. low sound-low action	45.662	11.470	3.981	**<0.001**	**0.003**
Action judgments: Low sound-high action vs. low sound-low action	-2.452	20.606	-0.119	0.906	1.000

Table S7. Study D (Martin et al. 2021): Repeated-measures ANOVA.

**Effect**	**df**	**F**	**p**	**partial η²**
**Error Rates**				
TASK	1,29	104.578	**<0.001**	**0.783**
DIFFICULTY	1,29	79.222	**<0.001**	**0.732**
TASK x DIFFICULTY	1,29	78.928	**<0.001**	**0.731**
**Mean RTs**				
TASK	1,29	62.868	**<0.001**	**0.684**
DIFFICULTY	1,29	63.783	**<0.001**	**0.687**
TASK x DIFFICULTY	1,29	44.498	**<0.001**	**0.605**

Table S8. Study D (Martin et al. 2021): Post-hoc paired t-tests.

**Comparison**	**Mean**	**SE**	**T**	**p (unc.)**	**p (corr.)**
**Error Rates**					
Semantic fluency vs. counting	7.900	0.773	10.226	**<0.001**	**<0.001**
Easy vs. difficult	-5.700	0.640	-8.901	**<0.001**	**<0.001**
Semantic fluency: easy vs. difficult	-11.067	1.200	9.226	**<0.001**	**<0.001**
Counting: easy vs. difficult	-0.333	0.333	1.000	0.326	0.326
**Mean RTs**					
Semantic fluency vs. counting	111.902	14.113	7.929	**<0.001**	**<0.001**
Easy vs. difficult	-56.620	7.090	-7.986	**<0.001**	**<0.001**
Semantic fluency: easy vs. difficult	-110.858	14.679	7.552	**<0.001**	**<0.001**
Counting: easy vs. difficult	-2.383	4.157	0.573	0.571	0.571

Table S9. Study E (Turker et al. 2021): Repeated-measures ANOVA.

**Effect**	**df**	**F**	**p**	**partial η²**
**Error Rates**				
WORD	1,27	33.582	**<0.001**	**0.554**
COMPLEXITY	1,27	30.878	**<0.001**	**0.533**
WORD x COMPLEXITY	1,27	32.220	**<0.001**	**0.544**
**Mean RTs**				
WORD	1,27	95.597	**<0.001**	**0.780**
COMPLEXITY	1,27	83.718	**<0.001**	**0.756**
WORD x COMPLEXITY	1,27	75.530	**<0.001**	**0.737**

Table S10. Study E (Turker et al. 2021): Post-hoc paired t-tests.

**Comparison**	**Mean**	**SE**	**T**	**p (unc.)**	**p (corr.)**
**Error Rates**					

Words vs. pseudowords	-6.071	1.048	-5.795	**<0.001**	**<0.001**
Complex vs. simple	4.071	0.733	5.557	**<0.001**	**<0.001**
Complex vs. simple words	-0.143	0.340	-0.420	0.678	0.678
Complex vs. simple pseudowords	8.286	1.435	5.772	**<0.001**	**<0.001**
Words vs. pseudowords for complex vs. simple	-8.429	1.485	-5.676	**<0.001**	**<0.001**
**Mean RTs**					
Words vs. pseudowords	-201.207	20.579	-9.777	**<0.001**	**<0.001**
Complex vs. simple	133.707	14.613	9.150	**<0.001**	**<0.001**
Complex vs. simple words	49.716	10.677	4.656	**<0.001**	**<0.001**
Complex vs. simple pseudowords	217.697	22.358	9.737	**<0.001**	**<0.001**
Words vs. pseudowords for complex vs. simple	-167.981	19.329	-8.691	**<0.001**	**<0.001**

ROI analyses for each study

The following tables report the results of statistical analyses on the response profiles of the four AG-ROIs in each study. ROI responses were estimated as percent signal change vs. the resting baseline. For statistical inference, we first conducted one-sample t-tests to identify significant activation or deactivation, as compared to the resting baseline, for each experimental condition. Then, we tested for relative activity differences between different experimental conditions via repeated-measures ANOVA. Significant effects were resolved using step-down analyses and post-hoc paired t-tests. Bold font highlights significant effects ($p < 0.05$ Bonferroni-Holm corrected for multiple comparisons); italic fonts highlights trends ($p < 0.05$ uncorrected).

Table S11. Study A (Chapman and Hartwigsen 2021): One sample t-tests vs. the resting baseline.

Task	Condition	Mean	SE	T	p (unc.)	p (corr.)
**Left PGa**						
Taxonomic task	Taxonomic	-0.034	0.015	-2.227	*0.032*	*0.095*
Taxonomic task	Thematic	0.002	0.013	0.133	0.895	1.000
Taxonomic task	Unrelated	-0.076	0.014	-5.635	**<0.001**	**<0.001**
Thematic task	Taxonomic	-0.049	0.011	-4.475	**<0.001**	**<0.001**
Thematic task	Thematic	-0.001	0.013	-0.069	0.945	1.000
Thematic task	Unrelated	-0.097	0.012	-7.984	**<0.001**	**<0.001**
Scrambled task	Congruent	-0.174	0.015	-11.325	**<0.001**	**<0.001**
Scrambled task	Incongruent	-0.114	0.016	-7.031	**<0.001**	**<0.001**
**Left PGp**						
Taxonomic task	Taxonomic	-0.003	0.019	-0.153	0.879	1.000
Taxonomic task	Thematic	0.033	0.016	2.017	0.050	0.252

Taxonomic task	Unrelated	0.006	0.014	0.402	0.690	1.000
Thematic task	Taxonomic	-0.013	0.012	-1.069	0.291	1.000
Thematic task	Thematic	0.052	0.016	3.304	**0.002**	**0.012**
Thematic task	Unrelated	0.007	0.013	0.504	0.617	1.000
Scrambled task	Congruent	-0.112	0.019	-5.846	**0.000**	**0.000**
Scrambled task	Incongruent	-0.074	0.019	-3.888	**0.000**	**0.003**
**Right PGa**						
Taxonomic task	Taxonomic	-0.047	0.015	-3.156	**0.003**	**0.012**
Taxonomic task	Thematic	-0.024	0.011	-2.125	*0.040*	*0.080*
Taxonomic task	Unrelated	-0.063	0.013	-4.671	**<0.001**	**<0.001**
Thematic task	Taxonomic	-0.039	0.012	-3.138	**0.003**	**0.012**
Thematic task	Thematic	-0.033	0.015	-2.113	*0.041*	*0.080*
Thematic task	Unrelated	-0.088	0.012	-7.560	**<0.001**	**<0.001**
Scrambled task	Congruent	-0.154	0.015	-10.361	**<0.001**	**<0.001**
Scrambled task	Incongruent	-0.103	0.013	-8.003	**<0.001**	**<0.001**
**Right PGp**						
Taxonomic task	Taxonomic	-0.006	0.018	-0.342	0.734	1.000
Taxonomic task	Thematic	0.014	0.015	0.951	0.347	1.000
Taxonomic task	Unrelated	-0.025	0.016	-1.488	0.145	0.723
Thematic task	Taxonomic	-0.002	0.014	-0.156	0.877	1.000
Thematic task	Thematic	0.015	0.017	0.888	0.380	1.000
Thematic task	Unrelated	-0.039	0.014	-2.786	*0.008*	*0.057*
Scrambled task	Congruent	-0.079	0.019	-4.224	**<0.001**	**0.001**
Scrambled task	Incongruent	-0.046	0.018	-2.535	*0.015*	*0.091*

Table S12. Study A (Chapman and Hartwigsen 2021): Repeated-measures ANOVA.

**Effect**	**df**	**F**	**p**	**partial η^2**
**Full ANOVA**				
ROI	3,120	13.447	**<0.001**	0.252
TASK	1,40	0.239	0.628	0.006
CONDITION	2,80	32.462	**<0.001**	0.448
ROI x TASK	3,120	1.138	0.330	0.028
ROI x CONDITION	6,240	29.120	**<0.001**	0.421
TASK x CONDITION	2,80	0.599	0.540	0.015
ROI x TASK x CONDITION	6,240	4.310	**0.001**	0.097

**Left PGa**				
TASK	1,40	0.931	0.340	0.023
CONDITION	2,80	66.395	**<0.001**	0.624
TASK x CONDITION	2,80	0.486	0.597	0.012
**Left PGp**				
TASK	1,40	0.077	0.783	0.002
CONDITION	2,80	20.109	**<0.001**	0.335
TASK x CONDITION	2,80	1.686	0.193	0.040
**Right PGa**				
TASK	1,40	0.534	0.469	0.013
CONDITION	2,80	20.165	**<0.001**	0.335
TASK x CONDITION	2,80	2.055	0.135	0.049
**Right PGp**				
TASK	1,40	0.069	0.794	0.002
CONDITION	2,80	21.422	**<0.001**	0.349
TASK x CONDITION	2,80	0.603	0.550	0.015

Table S13. Study A (Chapman and Hartwigsen 2021): Post-hoc paired t-tests.

**Comparison**	**Mean**	**SE**	**T**	**p (unc.)**	**p (corr.)**
**Left PGa**					
Taxonomic vs. thematic conditions	-0.042	0.006	-6.656	**<0.001**	**<0.001**
Taxonomic vs. unrelated conditions	0.045	0.008	5.593	**<0.001**	**<0.001**
Thematic vs. unrelated conditions	0.087	0.008	10.684	**<0.001**	**<0.001**
Taxonomic vs. thematic task	0.013	0.013	0.965	0.340	0.340
Taxonomic vs. scrambled task	0.107	0.016	6.858	**<0.001**	**<0.001**
Thematic vs. scrambled task	0.095	0.012	7.732	**<0.001**	**<0.001**
Scrambled task: congruent vs. incongruent	-0.060	0.011	-5.650	**<0.001**	**<0.001**
**Left PGp**					
Taxonomic vs. thematic conditions	-0.050	0.008	-6.678	**<0.001**	**<0.001**
Taxonomic vs. unrelated conditions	-0.014	0.009	-1.619	0.113	0.226
Thematic vs. unrelated conditions	0.036	0.008	4.438	**<0.001**	**<0.001**
Taxonomic vs. thematic task	-0.003	0.012	-0.278	0.783	0.783

Taxonomic vs. scrambled task	0.105	0.016	6.475	**<0.001**	**<0.001**
Thematic vs. scrambled task	0.108	0.014	7.622	**<0.001**	**<0.001**
Scrambled task: congruent vs. incongruent	-0.038	0.010	-3.847	**<0.001**	**<0.001**
**Right PGa**					
Taxonomic vs. thematic conditions	-0.015	0.007	-2.114	*0.041*	*0.082*
Taxonomic vs. unrelated conditions	0.033	0.008	4.192	**<0.001**	**<0.001**
Thematic vs. unrelated conditions	0.047	0.008	5.824	**<0.001**	**<0.001**
Taxonomic vs. thematic task	0.008	0.012	0.731	0.469	0.469
Taxonomic vs. scrambled task	0.084	0.015	5.409	**<0.001**	**<0.001**
Thematic vs. scrambled task	0.075	0.015	5.046	**<0.001**	**<0.001**
Scrambled task: congruent vs. incongruent	-0.051	0.010	-5.294	**<0.001**	**<0.001**
**Right PGp**					
Taxonomic vs. thematic conditions	-0.019	0.006	-3.009	**0.005**	**0.012**
Taxonomic vs. unrelated conditions	0.028	0.008	3.634	**0.001**	**0.005**
Thematic vs. unrelated conditions	0.046	0.007	6.210	**<0.001**	**<0.001**
Taxonomic vs. thematic task	0.003	0.012	0.263	0.794	0.794
Taxonomic vs. scrambled task	0.057	0.016	3.532	**0.001**	**0.005**
Thematic vs. scrambled task	0.054	0.014	3.953	**<0.001**	**<0.001**
Scrambled task: congruent vs. incongruent	-0.033	0.011	-3.057	**0.004**	**0.012**

Table S14. Study B (Graessner et al. 2021): One sample t-tests vs. the resting baseline.

Task	Condition	Mean	SE	T	p (unc.)	p (corr.)
**Left PGa**						
Implicit task	Meaningful phrases	-0.019	0.008	-2.379	*0.023*	*0.092*
Implicit task	Anomalous phrases	-0.019	0.007	-2.631	*0.013*	*0.065*
Implicit task	Pseudoword phrases	-0.037	0.009	-3.950	**<0.001**	**<0.001**
Implicit task	Single words	0.006	0.007	0.822	0.417	0.834
Explicit task	Meaningful phrases	0.001	0.006	0.181	0.857	0.857
Explicit task	Anomalous phrases	-0.020	0.007	-2.920	**0.006**	**0.036**
Explicit task	Pseudoword phrases	-0.033	0.006	-5.945	**<0.001**	**<0.001**
Explicit task	Single words	0.010	0.006	1.755	0.089	0.267
**Left PGp**						
Implicit task	Meaningful phrases	-0.033	0.007	-4.405	**<0.001**	**<0.001**
Implicit task	Anomalous phrases	-0.037	0.009	-4.257	**<0.001**	**<0.001**

Implicit task	Pseudoword phrases	-0.060	0.011	-5.388	**<0.001**	**<0.001**
Implicit task	Single words	-0.017	0.009	-1.996	0.054	0.054
Explicit task	Meaningful phrases	-0.018	0.007	-2.746	**0.010**	**0.020**
Explicit task	Anomalous phrases	-0.032	0.008	-4.167	**<0.001**	**<0.001**
Explicit task	Pseudoword phrases	-0.054	0.005	-10.377	**<0.001**	**<0.001**
Explicit task	Single words	-0.022	0.006	-3.516	**0.001**	**0.003**
**Right PGa**						
Implicit task	Meaningful phrases	-0.003	0.007	-0.461	0.648	0.894
Implicit task	Anomalous phrases	-0.009	0.006	-1.444	0.158	0.632
Implicit task	Pseudoword phrases	-0.009	0.011	-0.808	0.425	1.275
Implicit task	Single words	0.028	0.008	3.430	**0.002**	**0.016**
Explicit task	Meaningful phrases	-0.006	0.007	-0.771	0.447	1.275
Explicit task	Anomalous phrases	-0.017	0.007	-2.299	*0.028*	*0.168*
Explicit task	Pseudoword phrases	-0.011	0.006	-1.963	0.058	0.290
Explicit task	Single words	0.018	0.006	3.168	**0.003**	**0.021**
**Right PGp**						
Implicit task	Meaningful phrases	-0.035	0.006	-5.355	**<0.001**	**<0.001**
Implicit task	Anomalous phrases	-0.039	0.007	-5.417	**<0.001**	**<0.001**
Implicit task	Pseudoword phrases	-0.045	0.010	-4.348	**<0.001**	**<0.001**
Implicit task	Single words	-0.008	0.007	-1.090	0.284	0.284
Explicit task	Meaningful phrases	-0.028	0.009	-3.214	**0.003**	**0.009**
Explicit task	Anomalous phrases	-0.042	0.008	-5.454	**<0.001**	**<0.001**
Explicit task	Pseudoword phrases	-0.043	0.007	-6.659	**<0.001**	**<0.001**
Explicit task	Single words	-0.018	0.006	-3.005	**0.005**	**0.010**

Table S15. Study B (Graessner et al. 2021): Repeated-measures ANOVA.

**Effect**	**df**	**F**	**p**	**partial η^2**
**Full ANOVA**				
ROI	3,96	34.505	**<0.001**	0.519
TASK	1,32	0.054	0.818	0.002
CONDITION	3,96	30.543	**<0.001**	0.488
ROI x TASK	3,96	3.114	**0.032**	0.089
ROI x CONDITION	9,288	12.003	**<0.001**	0.273
TASK x CONDITION	3,96	1.714	0.183	0.051
ROI x TASK x CONDITION	9,288	2.020	**0.048**	0.059

**Left PGa**				
TASK	1,32	1.572	0.219	0.047
CONDITION	3,96	33.025	**<0.001**	0.508
TASK x CONDITION	3,96	3.472	**0.019**	0.098
**Left PGp**				
TASK	1,32	0.598	0.445	0.018
CONDITION	3,96	27.031	**<0.001**	0.458
TASK x CONDITION	3,96	2.060	0.114	0.060
**Right PGa**				
TASK	1,32	0.787	0.382	0.024
CONDITION	3,96	24.872	**<0.001**	0.437
TASK x CONDITION	3,96	0.511	0.629	0.016
**Right PGp**				
TASK	1,32	0.028	0.868	0.001
CONDITION	3,96	20.098	**<0.001**	0.386
TASK x CONDITION	3,96	1.480	0.233	0.044

Table S16. Study B (Graessner et al. 2021): Post-hoc paired t-tests.

**Comparison**	**Mean**	**SE**	**T**	**p (unc.)**	**p (corr.)**
**Left PGa**					
Implicit task: Meaningful vs. anomalous	0.000	0.004	0.045	0.965	1.000
Implicit task: Meaningful vs. pseudoword	0.019	0.006	3.197	**0.003**	**0.033**
Implicit task: Meaningful vs. single words	-0.025	0.005	-4.925	**<0.001**	**<0.001**
Implicit task: Anomalous vs. pseudoword	0.019	0.007	2.784	*0.009*	*0.081*
Implicit task: Anomalous vs. single words	-0.025	0.004	-5.859	**<0.001**	**<0.001**
Implicit task: Pseudoword vs. single words	-0.043	0.007	-6.013	**<0.001**	**<0.001**
Explicit task: Meaningful vs. anomalous	0.021	0.006	3.723	**0.001**	**0.012**
Explicit task: Meaningful vs. pseudoword	0.034	0.006	5.583	**<0.001**	**<0.001**
Explicit task: Meaningful vs. single words	-0.009	0.006	-1.548	0.132	0.528
Explicit task: Anomalous vs. pseudoword	0.013	0.006	2.389	*0.023*	*0.161*
Explicit task: Anomalous vs. single words	-0.030	0.005	-5.926	**<0.001**	**<0.001**
Explicit task: Pseudoword vs. single words	-0.043	0.006	-7.548	**<0.001**	**<0.001**

**Left PGp**					
Meaningful vs. anomalous phrases	0.009	0.005	1.747	0.090	0.180
Meaningful vs. pseudoword phrases	0.031	0.004	8.553	**<0.001**	**<0.001**
Meaningful phrases vs. single words	-0.006	0.005	-1.413	0.167	0.180
Anomalous vs. pseudoword phrases	0.023	0.005	4.517	**<0.001**	**<0.001**
Anomalous phrases vs. single words	-0.015	0.004	-3.883	**<0.001**	**<0.001**
Pseudowords phrases vs. single words	-0.038	0.005	-8.073	**<0.001**	**<0.001**
**Right PGa**					
Meaningful vs. anomalous phrases	0.009	0.004	2.068	*0.047*	*0.141*
Meaningful vs. pseudoword phrases	0.005	0.003	1.581	0.124	0.248
Meaningful phrases vs. single words	-0.027	0.005	-5.276	**<0.001**	**<0.001**
Anomalous vs. pseudoword phrases	-0.003	0.005	-0.681	0.501	0.501
Anomalous phrases vs. single words	-0.036	0.005	-7.493	**<0.001**	**<0.001**
Pseudowords phrases vs. single words	-0.033	0.005	-6.023	**<0.001**	**<0.001**
**Right PGp**					
Meaningful vs. anomalous phrases	0.009	0.004	2.266	*0.030*	*0.060*
Meaningful vs. pseudoword phrases	0.013	0.004	3.673	**0.001**	**0.004**
Meaningful phrases vs. single words	-0.019	0.005	-3.747	**0.001**	**0.004**
Anomalous vs. pseudoword phrases	0.003	0.004	0.831	0.412	0.412
Anomalous phrases vs. single words	-0.028	0.004	-6.518	**<0.001**	**<0.001**
Pseudowords phrases vs. single words	-0.032	0.005	-5.886	**<0.001**	**<0.001**

Table S17. Study C (Kuhnke et al. 2020): One sample t-tests vs. the resting baseline.

Task	Condition	Mean	SE	T	p (unc.)	p (corr.)
**Left PGa**						
Lexical decision	High sound, high action	-0.023	0.012	-1.855	0.071	0.712
Lexical decision	High sound, low action	-0.011	0.012	-0.891	0.378	1.000
Lexical decision	Low sound, high action	0.007	0.014	0.489	0.627	1.000
Lexical decision	Low sound, low action	-0.027	0.015	-1.853	0.072	0.712
Lexical decision	Pseudowords	-0.080	0.013	-6.301	**<0.001**	**<0.001**
Sound judgment	High sound, high action	0.027	0.014	1.974	0.056	0.611
Sound judgment	High sound, low action	0.020	0.016	1.251	0.218	1.000
Sound judgment	Low sound, high action	-0.012	0.011	-1.105	0.276	1.000
Sound judgment	Low sound, low action	-0.019	0.015	-1.320	0.194	1.000
Action judgment	High sound, high action	0.045	0.014	3.290	**0.002**	**0.026**

Action judgment	High sound, low action	0.007	0.013	0.538	0.594	1.000
Action judgment	Low sound, high action	0.019	0.015	1.219	0.230	1.000
Action judgment	Low sound, low action	-0.007	0.015	-0.479	0.634	1.000
**Left PGp**						
Lexical decision	High sound, high action	-0.006	0.013	-0.439	0.663	0.963
Lexical decision	High sound, low action	0.011	0.014	0.801	0.428	1.000
Lexical decision	Low sound, high action	0.015	0.015	0.995	0.326	1.000
Lexical decision	Low sound, low action	-0.011	0.016	-0.711	0.482	1.000
Lexical decision	Pseudowords	-0.056	0.014	-4.144	**<0.001**	0.002
Sound judgment	High sound, high action	0.038	0.014	2.692	*0.010*	*0.104*
Sound judgment	High sound, low action	0.042	0.014	2.930	*0.006*	*0.062*
Sound judgment	Low sound, high action	0.021	0.012	1.730	0.092	0.549
Sound judgment	Low sound, low action	0.024	0.015	1.606	0.116	0.581
Action judgment	High sound, high action	0.032	0.014	2.233	*0.031*	*0.219*
Action judgment	High sound, low action	0.032	0.014	2.320	*0.026*	*0.215*
Action judgment	Low sound, high action	0.036	0.015	2.352	*0.024*	*0.215*
Action judgment	Low sound, low action	0.041	0.013	3.069	**0.004**	**0.047**
**Right PGa**						
Lexical decision	High sound, high action	-0.009	0.013	-0.683	0.499	0.998
Lexical decision	High sound, low action	0.004	0.012	0.374	0.710	0.998
Lexical decision	Low sound, high action	0.020	0.013	1.522	0.136	0.900
Lexical decision	Low sound, low action	-0.016	0.012	-1.313	0.197	0.984
Lexical decision	Pseudowords	-0.042	0.011	-3.868	**<0.001**	**0.005**
Sound judgment	High sound, high action	-0.025	0.014	-1.780	0.083	0.662
Sound judgment	High sound, low action	-0.037	0.013	-2.765	*0.009*	*0.104*
Sound judgment	Low sound, high action	-0.019	0.012	-1.553	0.129	0.900
Sound judgment	Low sound, low action	-0.017	0.014	-1.283	0.207	0.984
Action judgment	High sound, high action	-0.015	0.013	-1.180	0.245	0.828
Action judgment	High sound, low action	-0.026	0.014	-1.874	0.068	0.631
Action judgment	Low sound, high action	-0.035	0.014	-2.455	*0.019*	*0.205*
Action judgment	Low sound, low action	-0.025	0.013	-1.913	0.063	0.631
**Right PGp**						
Lexical decision	High sound, high action	-0.031	0.011	-2.824	*0.007*	*0.089*
Lexical decision	High sound, low action	-0.006	0.011	-0.562	0.577	1.000

Lexical decision	Low sound, high action	-0.003	0.012	-0.219	0.828	1.000
Lexical decision	Low sound, low action	-0.028	0.012	-2.323	*0.026*	*0.230*
Lexical decision	Pseudowords	-0.053	0.011	-4.943	**<0.001**	**<0.001**
Sound judgment	High sound, high action	-0.020	0.012	-1.646	0.108	0.862
Sound judgment	High sound, low action	-0.030	0.012	-2.573	*0.014*	*0.140*
Sound judgment	Low sound, high action	-0.017	0.012	-1.462	0.152	1.000
Sound judgment	Low sound, low action	-0.012	0.013	-0.907	0.370	1.000
Action judgment	High sound, high action	-0.014	0.012	-1.145	0.259	1.000
Action judgment	High sound, low action	-0.018	0.012	-1.479	0.147	1.000
Action judgment	Low sound, high action	-0.034	0.013	-2.643	*0.012*	*0.130*
Action judgment	Low sound, low action	-0.015	0.012	-1.272	0.211	1.000

Table S18. Study C (Kuhnke et al. 2020): Repeated-measures ANOVA.

**Effect**	**df**	**F**	**p**	**partial η^2**
**Full ANOVA**				
ROI	3,117	10.429	**<0.001**	0.211
TASK	2,78	0.327	0.705	0.008
SOUND	1,39	1.353	0.252	0.034
ACTION	1,39	1.515	0.226	0.037
ROI x TASK	6,234	12.386	**<0.001**	0.241
ROI x SOUND	3,117	12.536	**<0.001**	0.243
TASK x SOUND	2,78	1.040	0.358	0.026
ROI x TASK x SOUND	6,234	9.546	**<0.001**	0.197
ROI x ACTION	3,117	6.650	**<0.001**	0.146
TASK x ACTION	2,78	0.102	0.900	0.003
ROI x TASK x ACTION	6,234	2.580	**0.028**	0.062
SOUND x ACTION	1,39	1.577	0.217	0.039
ROI x SOUND x ACTION	3,117	0.716	0.542	0.018
TASK x SOUND x ACTION	2,78	4.345	**0.019**	0.100
ROI x TASK x SOUND x ACTION	6,234	0.577	0.738	0.015
**Left PGa**				
TASK	2,78	3.058	0.055	0.073
SOUND	1,39	12.946	**0.001**	0.249
ACTION	1,39	9.376	**0.004**	0.194
TASK x SOUND	2,78	7.104	**0.001**	0.154
TASK x ACTION	2,78	2.253	0.112	0.055

SOUND x ACTION	2,78	3.058	0.055	0.073
**Left PGp**				
TASK	2,78	4.365	**0.021**	0.101
SOUND	1,39	0.735	0.397	0.018
ACTION	1,39	0.007	0.933	0.000
TASK x SOUND	2,78	2.324	0.105	0.056
TASK x ACTION	2,78	0.347	0.705	0.009
SOUND x ACTION	2,78	4.365	**0.021**	0.101
**Right PGa**				
TASK	2,78	2.809	0.071	0.067
SOUND	1,39	0.411	0.525	0.010
ACTION	1,39	1.487	0.230	0.037
TASK x SOUND	2,78	2.043	0.137	0.050
TASK x ACTION	2,78	0.431	0.652	0.011
SOUND x ACTION	2,78	2.809	0.071	0.067
**Right PGp**				
TASK	2,78	0.054	0.947	0.001
SOUND	1,39	0.318	0.576	0.008
ACTION	1,39	0.140	0.710	0.004
TASK x SOUND	2,78	2.038	0.137	0.050
TASK x ACTION	2,78	0.381	0.666	0.010
SOUND x ACTION	2,78	0.054	0.947	0.001

Table S19. Study C (Kuhnke et al. 2020): Post-hoc paired t-tests.

Comparison	Mean	SE	T	p (unc.)	p (corr.)
**Left PGa**					
Lexical decision: Words vs. Pseudowords	0.067	0.008	8.021	**<0.001**	**<0.001**
Lexical decision: High- vs. low-sound words	-0.006	0.009	-0.745	0.461	0.922
Lexical decision: High- vs. low-action words	0.011	0.008	1.393	0.171	0.513
Sound judgment: High- vs. low-sound words	0.039	0.009	4.424	**<0.001**	**<0.001**
Sound judgment: High- vs. low-action words	0.007	0.009	0.732	0.469	0.922
Sound judgment: High- vs. low-action words	0.032	0.010	3.161	**0.003**	**0.015**
Sound judgment: High- vs. low-sound words	0.021	0.008	2.446	*0.019*	*0.076*

**Left PGp**					
Lexical decision: Words vs. Pseudowords	0.058	0.008	7.210	**<0.001**	**<0.001**
Lexical decisions vs. Sound judgments	-0.029	0.012	-2.376	*0.023*	*0.069*
Lexical decisions vs. Action judgments	-0.033	0.014	-2.289	*0.028*	*0.069*
Sound judgments vs. Action judgments	-0.004	0.009	-0.428	0.671	0.671
**Right PGa**					
Lexical decision: Words vs. Pseudowords	0.041	0.009	4.635	**<0.001**	**<0.001**
Lexical decision: High- vs. low-sound words	-0.004	0.007	-0.571	0.571	1.000
Lexical decision: High- vs. low-action words	0.011	0.008	1.440	0.158	0.858
Sound judgment: High- vs. low-sound words	-0.013	0.009	-1.496	0.143	0.858
Sound judgment: High- vs. low-action words	0.005	0.008	0.625	0.536	1.000
Sound judgment: High- vs. low-action words	0.000	0.009	0.045	0.964	1.000
Sound judgment: High- vs. low-sound words	0.009	0.007	1.338	0.189	0.790
**Right PGp**					
Lexical decision: Words vs. Pseudowords	0.036	0.007	5.442	**<0.001**	**<0.001**
Lexical decision: High- vs. low-sound words	-0.004	0.007	-0.539	0.593	1.000
Lexical decision: High- vs. low-action words	0.000	0.008	-0.010	0.992	1.606
Sound judgment: High- vs. low-sound words	-0.011	0.007	-1.557	0.128	0.768
Sound judgment: High- vs. low-action words	0.002	0.008	0.251	0.803	1.000
Sound judgment: High- vs. low-action words	-0.007	0.008	-0.911	0.368	1.000
Sound judgment: High- vs. low-sound words	0.008	0.006	1.374	0.177	0.885

Table S20. Study D (Martin et al. 2021): One sample t-tests vs. the resting baseline.

**Task**	**Condition**	**Mean**	**SE**	**T**	**p (unc.)**	**p (corr.)**
**Left PGa**						
Semantic fluency	Difficult	-0.367	0.036	-10.077	**<0.001**	**<0.001**
Semantic fluency	Easy	-0.285	0.033	-8.716	**<0.001**	**<0.001**
Counting	Difficult	-0.233	0.038	-6.182	**<0.001**	**<0.001**
Counting	Easy	-0.188	0.033	-5.650	**<0.001**	**<0.001**
**Left PGp**						
Semantic fluency	Difficult	-0.265	0.031	-8.416	**<0.001**	**<0.001**
Semantic fluency	Easy	-0.196	0.031	-6.390	**<0.001**	**<0.001**
Counting	Difficult	-0.202	0.030	-6.841	**<0.001**	**<0.001**
Counting	Easy	-0.139	0.023	-5.969	**<0.001**	**<0.001**

**Right PGa**						
Semantic fluency	Difficult	-0.428	0.053	-8.072	**<0.001**	**<0.001**
Semantic fluency	Easy	-0.298	0.045	-6.563	**<0.001**	**<0.001**
Counting	Difficult	-0.155	0.028	-5.621	**<0.001**	**<0.001**
Counting	Easy	-0.160	0.034	-4.753	**<0.001**	**<0.001**
**Right PGp**						
Semantic fluency	Difficult	-0.494	0.052	-9.465	**<0.001**	**<0.001**
Semantic fluency	Easy	-0.311	0.040	-7.703	**<0.001**	**<0.001**
Counting	Difficult	-0.188	0.024	-7.840	**<0.001**	**<0.001**
Counting	Easy	-0.162	0.026	-6.345	**<0.001**	**<0.001**

Table S21. Study D (Martin et al. 2021): Repeated-measures ANOVA.

**Effect**	**df**	**F**	**p**	**partial η^2**
**Full ANOVA**				
ROI	3,87	8.243	**<0.001**	0.221
TASK	1,29	23.292	**<0.001**	0.445
DIFFICULTY	1,29	8.691	**0.006**	0.231
ROI x TASK	3,87	9.901	**<0.001**	0.255
ROI x DIFFICULTY	3,87	3.944	**0.013**	0.120
TASK x DIFFICULTY	1,29	6.571	**0.016**	0.185
ROI x TASK x DIFFICULTY	3,87	5.446	**0.008**	0.158
**Left PGa**				
TASK	1,29	8.334	**0.007**	0.223
DIFFICULTY	1,29	7.346	**0.011**	0.202
TASK x DIFFICULTY	1,29	0.733	0.399	0.025
**Left PGp**				
TASK	1,29	4.268	**0.048**	0.128
DIFFICULTY	1,29	7.958	**0.009**	0.215
TASK x DIFFICULTY	1,29	0.029	0.865	0.001
**Right PGa**				
TASK	1,29	27.069	**<0.001**	0.483
DIFFICULTY	1,29	4.893	**0.035**	0.144

TASK x DIFFICULTY	1,29	6.410	**0.017**	0.181
**Right PGp**				
TASK	1,29	28.245	**<0.001**	0.493
DIFFICULTY	1,29	11.589	**0.002**	0.286
TASK x DIFFICULTY	1,29	20.217	**<0.001**	0.411

Table S22. Study D (Martin et al. 2021): Post-hoc paired t-tests.

Comparison	Mean	SE	T	p (unc.)	p (corr.)
**Left PGa**					
Semantic fluency vs. counting	-0.115	0.040	-2.887	**0.007**	**0.014**
Easy vs. difficult	0.064	0.024	2.710	**0.011**	**0.011**
**Left PGp**					
Semantic fluency vs. counting	-0.060	0.029	-2.066	**0.048**	**0.048**
Easy vs. difficult	0.066	0.023	2.821	**0.009**	**0.018**
**Right PGa**					
Semantic fluency vs. counting	-0.206	0.040	-5.203	**<0.001**	**<0.001**
Easy vs. difficult	0.063	0.028	2.212	*0.035*	*0.070*
Semantic fluency: easy vs. difficult	0.130	0.046	2.851	**0.008**	**0.024**
Counting: easy vs. difficult	-0.004	0.031	-0.132	0.896	0.896
**Right PGp**					
Semantic fluency vs. counting	-0.228	0.043	-5.315	**<0.001**	**<0.001**
Easy vs. difficult	0.105	0.031	3.404	**0.002**	**0.004**
Semantic fluency: easy vs. difficult	0.183	0.039	4.668	**<0.001**	**<0.001**
Counting: easy vs. difficult	0.027	0.031	0.857	0.399	0.399

Table S23. Study E (Turker et al. 2021): One sample t-tests vs. the resting baseline.

Condition	Mean	SE	T	p (unc.)	p (corr.)
**Left PGa**					
Complex words	-0.025	0.021	-1.223	0.232	0.232
Simple words	0.029	0.017	1.705	0.100	0.199
Complex pseudowords	-0.235	0.028	-8.525	**<0.001**	**<0.001**
Simple pseudowords	-0.075	0.020	-3.670	**0.001**	**0.003**

**Left PGp**					
Complex words	-0.019	0.019	-0.960	0.345	0.460
Simple words	0.016	0.013	1.229	0.230	0.460
Complex pseudowords	-0.159	0.027	-5.826	**<0.001**	**<0.001**
16Simple pseudowords	-0.080	0.019	-4.227	**<0.001**	**0.001**
**Right PGa**					
Complex words	-0.044	0.022	-2.002	0.055	0.111
Simple words	-0.009	0.016	-0.561	0.579	0.579
Complex pseudowords	-0.250	0.028	-8.826	**<0.001**	**<0.001**
Simple pseudowords	-0.097	0.027	-3.539	**0.001**	**0.004**
**Right PGp**					
Complex words	-0.054	0.018	-3.053	**0.005**	**0.010**
Simple words	-0.019	0.015	-1.236	0.227	0.227
Complex pseudowords	-0.198	0.027	-7.286	**<0.001**	**<0.001**
Simple pseudowords	-0.096	0.021	-4.670	**<0.001**	**<0.001**

Table S24. Study E (Turker et al. 2021): Repeated-measures ANOVA.

**Effect**	**df**	**F**	**p**	**partial η^2**
**Full ANOVA**				
ROI	3,81	2.892	**0.049**	0.097
WORD	1,27	132.208	**<0.001**	0.830
ROI x WORD	3,81	4.087	**0.018**	0.131
ROI x COMPLEXITY	3,81	10.073	**<0.001**	0.272
WORD x COMPLEXITY	1,27	10.034	**0.004**	0.271
ROI x WORD x COMPLEXITY	3,81	6.926	**0.001**	0.204
**Left PGa**				
WORD	1,27	95.790	**<0.001**	0.780
COMPLEXITY	1,27	58.472	**<0.001**	0.684
WORD x COMPLEXITY	1,27	14.446	**0.001**	0.349
**Left PGp**				
WORD	1,27	110.783	**<0.001**	0.804
COMPLEXITY	1,27	16.148	**<0.001**	0.374
WORD x COMPLEXITY	1,27	3.484	0.073	0.114

**Right PGa**				
WORD	1,27	72.858	**<0.001**	0.730
COMPLEXITY	1,27	52.889	**<0.001**	0.662
WORD x COMPLEXITY	1,27	14.596	**0.001**	0.351
**Right PGp**				
WORD	1,27	57.379	**<0.001**	0.680
COMPLEXITY	1,27	23.459	**<0.001**	0.465
WORD x COMPLEXITY	1,27	4.441	**0.045**	0.141

Table S25. Study E (Turker et al. 2021): Post-hoc paired t-tests.

Comparison	Mean	SE	T	p (unc.)	p (corr.)
**Left PGa**					
Words vs. pseudowords	0.157	0.016	9.787	**<0.001**	**<0.001**
Complex vs. simple	-0.108	0.014	-7.647	**<0.001**	**<0.001**
Complex vs. simple words	-0.054	0.016	-3.499	**0.002**	**0.002**
Complex vs. simple pseudowords	-0.161	0.023	-6.876	**<0.001**	**<0.001**
Words vs. pseudowords for complex vs. simple	0.106	0.028	3.801	**0.001**	**0.002**
**Left PGp**					
Words vs. pseudowords	0.118	0.011	10.525	**<0.001**	**<0.001**
Complex vs. simple	-0.057	0.014	-4.018	**<0.001**	**<0.001**
Complex vs. simple words	-0.035	0.014	-2.467	**0.020**	**0.040**
Complex vs. simple pseudowords	-0.079	0.022	-3.583	**0.001**	**0.003**
Words vs. pseudowords for complex vs. simple	0.045	0.024	1.866	0.073	0.073
**Right PGa**					
Words vs. pseudowords	0.147	0.017	8.536	**<0.001**	**<0.001**
Complex vs. simple	-0.094	0.013	-7.272	**<0.001**	**<0.001**
Complex vs. simple words	-0.035	0.020	-1.791	0.085	0.085
Complex vs. simple pseudowords	-0.153	0.021	-7.371	**<0.001**	**<0.001**
Words vs. pseudowords for complex vs. simple	0.118	0.031	3.820	**0.001**	**0.002**
**Right PGp**					
Words vs. pseudowords	0.111	0.015	7.575	**<0.001**	**<0.001**
Complex vs. simple	-0.068	0.014	-4.843	**<0.001**	**<0.001**

Complex vs. simple words	-0.035	0.017	-2.067	*0.048*	*0.090*
Complex vs. simple pseudowords	-0.102	0.025	-4.083	**<0.001**	**<0.001**
Words vs. pseudowords for complex vs. simple	0.067	0.032	2.107	*0.045*	*0.090*

Linear-mixed-model analysis across all studies

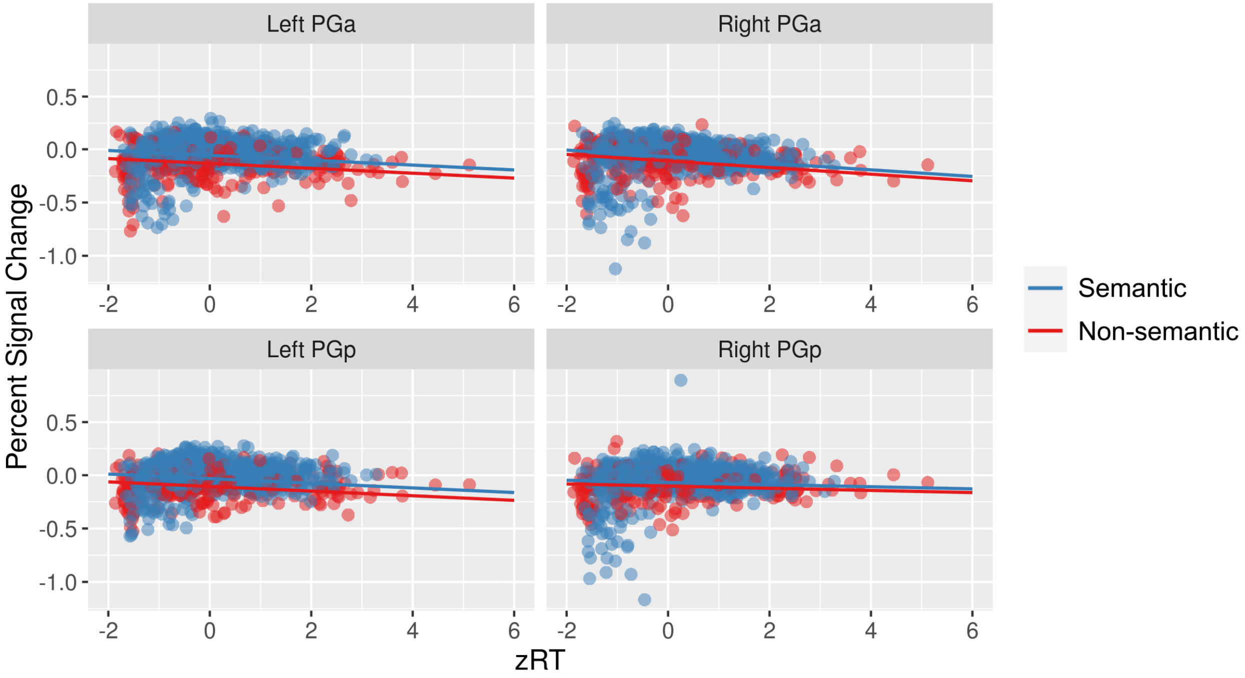

Figure S1. Results of the linear-mixed-model analysis. Combined effects of semantics and zRT on predicted percent signal change in each ROI, including raw data points.

Table S26. ANOVA table of the next best-fitting model that included the zRT x Semantics interaction.

Factor	SS	MSE	DF	Den. DF	F	p
zRT	0.32	0.32	1	4876.6	39.63	**< 0.001**
Sem	0.09	0.09	1	27.9	10.96	**0.003**
ROI	0.35	0.12	3	5187.4	14.67	**< 0.001**
zRT x ROI	0.29	0.10	3	5187.4	12.18	**< 0.001**
Sem x ROI	0.33	0.11	3	5187.4	13.68	**< 0.001**
zRT x Sem	0.01	0.01	1	5006.1	1.69	0.194

SS = sum of squares; MSE = mean squared error; DF = degrees of freedom; Den = denominator; Sem = effect of semantics.

Table S27. Fixed effects of mixed model with PGa vs. PGp and left vs. right contrasts.

	Estimate	SE	df	t	p
Intercept	-0.085	0.031	11.6	-2.74	**0.018**
zRT	-0.021	0.003	4812.7	-6.74	**<0.001**
Sem vs. Non-sem	0.056	0.017	27.7	3.39	**0.002**
ROI: PGa vs. PGp	-0.031	0.006	5187.3	-5.22	**<0.001**
ROI: Left vs. Right	0.011	0.006	5187.3	1.84	0.066
zRT x PGa vs. PGp	-0.022	0.005	5187.3	-4.44	**<0.001**
zRT x Left vs. Right	-0.004	0.005	5187.3	-0.72	0.470
Sem vs. Non-sem x PGa vs. PGp	0.009	0.012	5187.3	0.74	0.460
Sem vs. Non-sem x Left vs. Right	0.075	0.012	5187.3	6.33	**<0.001**

Table S28. Fixed effects of left PGa model.

	Estimate	SE	df	t	p
Intercept	-0.13	0.033	16.4	-3.94	**0.001**
zRT	-0.01	0.006	1071.1	-2.43	**0.015**
Sem vs. Non-sem	0.07	0.021	29.1	3.53	**0.001**

Table S29. Fixed effects of left PGp model.

	Estimate	SE	df	t	p
Intercept	-0.09	0.028	13.9	-3.32	**0.005**
zRT	-0.02	0.005	1108.9	-4.75	**<0.001**
Sem vs. Non-sem	0.06	0.013	27.5	4.51	**<0.001**

Table S30. Fixed effects of right PGa model.

	Estimate	SE	df	t	p
Intercept	-0.12	0.036	14.6	-3.23	**0.006**
zRT	-0.03	0.006	1189.2	-4.60	**<0.001**
Sem vs. Non-sem	0.05	0.019	27.3	2.68	**0.012**

Table S31. Fixed effects of right PGp model.

	Estimate	SE	df	t	p
Intercept	-0.11	0.040	13.6	-2.88	**0.013**
zRT	-0.02	0.006	1230.3	-3.71	**<0.001**
Sem vs. Non-sem	0.04	0.018	26.8	2.45	**0.021**

Separate linear-mixed-model analyses for semantic and non-semantic conditions

To test whether RT effects in the AG are indeed domain-general and emerge in both semantic and non-semantic conditions, we ran two additional linear-mixed-model analyses for semantic and non-semantic conditions separately.

The results show that the optimal model of AG responses included main effects of zRT and ROI, as well as interactions of zRT x ROI for both semantic conditions (Tables S32-S33) and non-semantic conditions (Tables S34-S35). These findings support our conclusion that effects of RT on AG activity are domain-general and independent of semantics.

Semantic conditions only

Table S32. AIC comparisons to determine the optimal model predicting AG activity for semantic conditions only.

Comparison	Model	ΔAIC	df	
**(A) Random effects**	**PSC ~ 1 + (1 | participant) + (1 | task/condition)**	**0**	**5**	*
	PSC ~ 1 + (1 | participant) + (1 | condition)	18.4	4	
	PSC ~ 1 + (1 | participant) + (1 | task)	140.4	4	
	PSC ~ 1 + (1 | participant)	985.9	3	
**(B) zRT & zER**	**PSC ~ zRT + (1 | participant) + (1 | task/condition)**	**0**	**6**	*
	PSC ~ zER + (1 | participant) + (1 | task/condition)	28.9	6	
	PSC ~ 1 + (1 | participant) + (1 | task/condition)	32.0	5	
**(C) Interactions**	**PSC ~ zRT + ROI + zRT:ROI + (1 | participant) + (1 | task/condition)**	**0**	**12**	*
	PSC ~ zRT + ROI + (1 | participant) + (1 | task/condition)	20.3	9	
	PSC ~ zRT + (1 | participant) + (1 | task/condition)	140.3	6	

* = winning model; PSC = percent signal change; zRT = standardized RT; zER = standardized error rate; AIC = Akaike Information Criterion.

Table S33. ANOVA table for the optimal model of semantic conditions.

Factor	SS	MSE	DF	Den. DF	F	p
zRT	0.22	0.22	1	3821.3	30.33	**< 0.001**
ROI	0.91	0.30	3	3983.8	42.70	**< 0.001**
zRT x ROI	0.19	0.06	3	3983.8	8.80	**< 0.001**

SS = sum of squares; MSE = mean squared error; DF = degrees of freedom; Den = denominator.

Non-semantic conditions only

Table S34. AIC comparisons to determine the optimal model predicting AG activity for non-semantic conditions only.

Comparison	Model	ΔAIC	df	
**(A) Random effects**	**PSC ~ 1 + (1 | participant) + (1 | condition)**	**0**	**4**	*
	PSC ~ 1 + (1 | participant) + (1 | task/condition)	6.6	5	
	PSC ~ 1 + (1 | participant) + (1 | task)	106.8	4	
	PSC ~ 1 + (1 | participant)	252.6	3	
**(B) zRT & zER**	**PSC ~ zRT + (1 | participant) + (1 | condition)**	**0**	**5**	*
	PSC ~ zER + (1 | participant) + (1 | task/condition)	11.1	5	
	PSC ~ 1 + (1 | participant) + (1 | task/condition)	14.7	4	
**(C) Interactions**	**PSC ~ zRT + ROI + zRT:ROI + (1 | participant) + (1 | condition)**	**0**	**11**	*
	PSC ~ zRT + ROI + (1 | participant) + (1 | condition)	7.6	8	
	PSC ~ zRT + (1 | participant) + (1 | condition)	23.3	5	

* = winning model; PSC = percent signal change; zRT = standardized RT; zER = standardized error rate; AIC = Akaike Information Criterion.

Table S35. ANOVA table for the optimal model of non-semantic conditions.

Factor	SS	MSE	DF	Den. DF	F	p
zRT	0.10	0.10	1	763.7	12.14	**< 0.001**
ROI	0.17	0.06	3	1084.8	6.86	**< 0.001**
zRT x ROI	0.11	0.04	3	1084.8	4.55	**0.004**

SS = sum of squares; MSE = mean squared error; DF = degrees of freedom; Den = denominator.

Effect of semantics on RT

To investigate the relationship between the semantics and RT variables in our model, we ran a supplementary linear-mixed-model analysis that aimed to explain zRT by semantics and random effects as in our original model (i.e. subject and condition nested within task):

zRT ~ Sem + (1 | participant) + (1 | task/condition)

The results show that the effect of semantics on zRT is significantly different from zero (Table S36), but very small, explaining only 1.1% of the variance in RT (Table S37, R^2 marg.). Note that the entire model explains 89.1% of the variance in RT (Table S37, R^2 cond.).

Table S36. ANOVA table of the linear-mixed-effects model explaining zRT by semantics.

Factor	SS	MSE	DF	Den. DF	F	p
Sem	0.81	0.81	1	26.16	5.69	**0.025**

SS = sum of squares; MSE = mean squared error; DF = degrees of freedom; Den = denominator; Sem = effect of semantics.

Table S37. Performance indices of the linear-mixed-effects model explaining zRT by semantics.

AIC	BIC	R^2 cond.	R^2 (marg.)	ICC	RMSE
5356.009	5395.511	0.891	**0.011**	0.889	0.371

AIC = Akaike information criterion; BIC = Bayesian information criterion; R^2 cond. = proportion of variance explained by fixed and random effects**;** R^2 marg. = proportion of variance explained by fixed effects (Sem) only; ICC = Intraclass Correlation Coefficient; RMSE = Root Mean Squared Error.

Implicit vs. explicit semantic tasks

In our main linear-mixed-model analysis, the semantics variable parsimoniously distinguished semantic and non-semantic experimental conditions. Arguably, semantic conditions can be subdivided into *explicit* semantic conditions (when the task explicitly requires the retrieval of semantic information, e.g. in semantic judgments) and *implicit* semantic conditions (when the task only implicitly probes semantic processing, e.g. in lexical decisions). Therefore, we performed a supplementary linear-mixed-model analysis, where we compared our original "semantics" (semantic vs. non-semantic) model against a "semanticity" (explicit semantic vs. implicit semantic vs. non-semantic) model.

The results show that the "semanticity" model is not better than the "semantics" model (Table S38). In fact, the "semanticity" model is worse (i.e., has a higher AIC) as both models have the same log-likelihood but the "semantics" model is less complex (1 fewer degree of freedom). Therefore, our main "semantics" model remains the optimal model of AG responses across the five studies. Note, however, that it is questionable whether the "semanticity" model is a valid model for our data as only two of the five studies (studies B and C) included both implicit and explicit semantic tasks.

Table S38. AIC comparisons to determine the optimal model predicting AG activity.

**Model**	**ΔLogLik**	**ΔAIC**	**df**	
**PSC ~ Semantics + (1 | participant) + (1 | task/condition)**	**5.2**	**0**	**6**	*
PSC ~ Semanticity + (1 | participant) + (1 | condition)	5.2	2.0	7	
PSC ~ 1 + (1 | participant) + (1 | task/condition)	0	8.4	5	

* = winning model; PSC = percent signal change; LogLik: log-likelihood; AIC = Akaike Information Criterion; df = degrees of freedom.
